# Reversal of ciliary mechanisms of disassembly rescues olfactory dysfunction in ciliopathies

**DOI:** 10.1172/jci.insight.158736

**Published:** 2022-08-08

**Authors:** Chao Xie, Julien C. Habif, Kirill Ukhanov, Cedric R. Uytingco, Lian Zhang, Robert J. Campbell, Jeffrey R. Martens

**Affiliations:** 1 Department of Pharmacology and Therapeutics and; 2 Center for Smell and Taste, University of Florida College of Medicine, Gainesville, Florida, USA.

**Keywords:** Cell Biology, Genetics, Gene therapy, Genetic diseases, Mouse models

## Abstract

Ciliopathies are a class of genetic diseases resulting in cilia dysfunction in multiple organ systems, including the olfactory system. Currently, there are no available curative treatments for olfactory dysfunction and other symptoms in ciliopathies. The loss or shortening of olfactory cilia, as seen in multiple mouse models of the ciliopathy Bardet–Biedl syndrome (BBS), results in olfactory dysfunction. However, the underlying mechanism of the olfactory cilia reduction is unknown, thus limiting the development of therapeutic approaches for BBS and other ciliopathies. Here, we demonstrated that phosphatidylinositol 4,5-bisphosphate [PI(4,5)P_2_], a phosphoinositide typically excluded from olfactory cilia, aberrantly redistributed into the residual cilia of BBS mouse models, which caused F-actin ciliary infiltration. Importantly, PI(4,5)P_2_ and F-actin were necessary for olfactory cilia shortening. Using a gene therapeutic approach, the hydrolyzation of PI(4,5)P_2_ by overexpression of inositol polyphosphate-5-phosphatase E (INPP5E) restored cilia length and rescued odor detection and odor perception in BBS. Together, our data indicate that PI(4,5)P_2_/F-actin–dependent cilia disassembly is a common mechanism contributing to the loss of olfactory cilia in BBS and provide valuable pan-therapeutic intervention targets for the treatment of ciliopathies.

## Graphical abstract

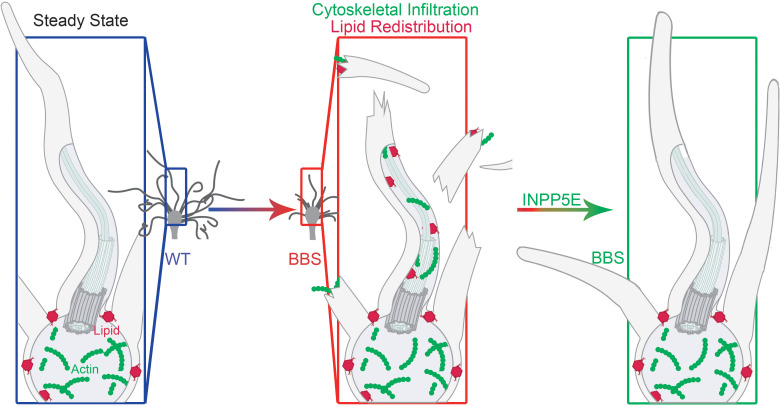

## Introduction

Cilia are evolutionarily conserved, microtubule-based organelles that are present on the surface of most cell types in vertebrates (1). The enrichment of various receptors and other ciliary exclusive proteins (2, 3) makes the cilium a unique organelle with critical roles in numerous developmental and fundamental physiological processes (4–6). Genetic defects of ciliary proteins that are necessary for cilia biogenesis, maintenance, and/or function can result in a broad class of human diseases and developmental disorders, termed ciliopathies (7). As a class of ciliopathies, Bardet–Biedl syndrome (BBS) can manifest as a constellation of symptoms including obesity, renal dysfunction, male infertility, skeletal malformation, cognitive defects, and retinal degeneration (8–10). Furthermore, BBS has been characterized as a major genetic cause of olfactory dysfunction (9–11), which is a relatively common disorder (12) that markedly decreases the quality of life and increases the risk of injuries (13). Besides symptom management, there are no curative treatment options currently available for BBS and other ciliopathies. Several preclinical studies have shown that single-gene replacement is a promising curative therapeutic approach for olfactory dysfunction in ciliopathies (9, 10, 14); however, it is limited to the treatment of only a small subset of patients with genetic mutations in the targeted gene (15).

BBS is caused by 1 or more mutation(s) in any of at least 21 proteins related to the BBSome, which is a highly conserved complex comprising 8 core BBS proteins: BBS1, BBS2, BBS4, BBS5, BBS7, BBS8, BBS9, and BBS18/BBIP10 (16, 17). The BBSome mainly functions as a cargo adaptor for intraflagellar transport (IFT) that regulates protein ciliary trafficking (16, 18, 19). The mutation or deletion of BBSome-related genes typically alters morphology, length, and dynamics of cilia in different organ systems with diverse effects on cilium maintenance (9–11, 20). In the olfactory system, olfactory cilia extend from olfactory sensory neurons (OSNs), providing a large odorant-receptive field (5, 21). Defects in olfactory cilia, as seen in animal models of BBS, lead to significant impairment (hyposmia) or complete loss (anosmia) of olfactory function (9, 10, 14, 22). Studies have shown that BBS mouse models share similar olfactory phenotypes, specifically with decreased olfactory cilia length and number (9–11, 23, 24), suggesting that a shared mechanism may contribute to the pathogenesis of BBS. However, the detailed mechanism of olfactory cilia reduction in BBS has not been determined; the understanding of the mechanism may suggest novel therapeutic targets for BBS.

In normal conditions, cilia are dynamic structures with a tightly regulated balance among cilia formation, maintenance, and disassembly (25, 26). Different ciliopathies can be caused by dysfunction in any of these 3 ciliary processes. In the context of BBS, olfactory cilia are reduced but not completely lost, and the residual cilia still have a persistent trafficking of both IFT and protein, which is essential for cilia maintenance (9, 10). These indicate that olfactory cilia formation and maintenance may not be affected in BBS. Importantly, there is a progressive loss of olfactory cilia resulting from the deletion of BBS4 (9). Together, these pieces of evidence suggest that olfactory cilia form and are maintained properly but that olfactory cilia disassembly may contribute to the loss of cilia in BBS. A recent study in vitro in cultured cells showed that the normal process of primary cilia resorption that occurs during the cell cycle involves membrane-composition remolding that contributes to primary cilia disassembly (27). The membrane composition in cilia differs from that of the cellular membrane (28–31). For instance, phosphatidylinositol 4,5-bisphosphate [PI(4,5)P_2_], a phospholipid component of the cell membrane, is enriched at the base of cilia but is excluded from the ciliary membrane because of the presence of its hydrolase, inositol polyphosphate-5-phosphatase E (INPP5E) (32–35). In culture, growth stimulation of quiescent cells results in the accumulation of PI(4,5)P_2_ in primary cilia and promotes cilia disassembly (27). Importantly, BBS5 contains 2 lipid-binding pleckstrin homology–like (PH-like) domains, which facilitate the direct interaction between the BBSome and phospholipids (16). Furthermore, BBS4 is implicated in regulating the ciliary distribution of INPP5E in primary cilia (36), suggesting that the BBSome plays important roles in the regulation of the ciliary membrane composition. However, all of these studies were conducted in cultured, dividing cells in primary cilia, which are remarkably different from olfactory cilia in the terminally differentiated neurons (5, 21). It is largely unclear if such lipid remolding can take place in olfactory cilia in vivo or if it also contributes to the pathogenesis of BBS disease in the olfactory system. In this study, we investigate the signaling mechanisms underlying the loss or shortening of olfactory cilia under pathological conditions in BBS-mutant mice. Importantly, using a gene therapeutic approach, we demonstrate that olfactory dysfunction in BBS can be rescued by targeting a common factor that regulates the ciliary length. This mechanistic study highlights alternative therapeutic targets for treating ciliary dysfunction in ciliopathies, which may allow gene therapy to move beyond single-gene replacement.

## Results

### PI(4,5)P_2_ aberrantly redistributes into residual olfactory cilia in Bbs4^KO^ mice.

**Figure 1:**
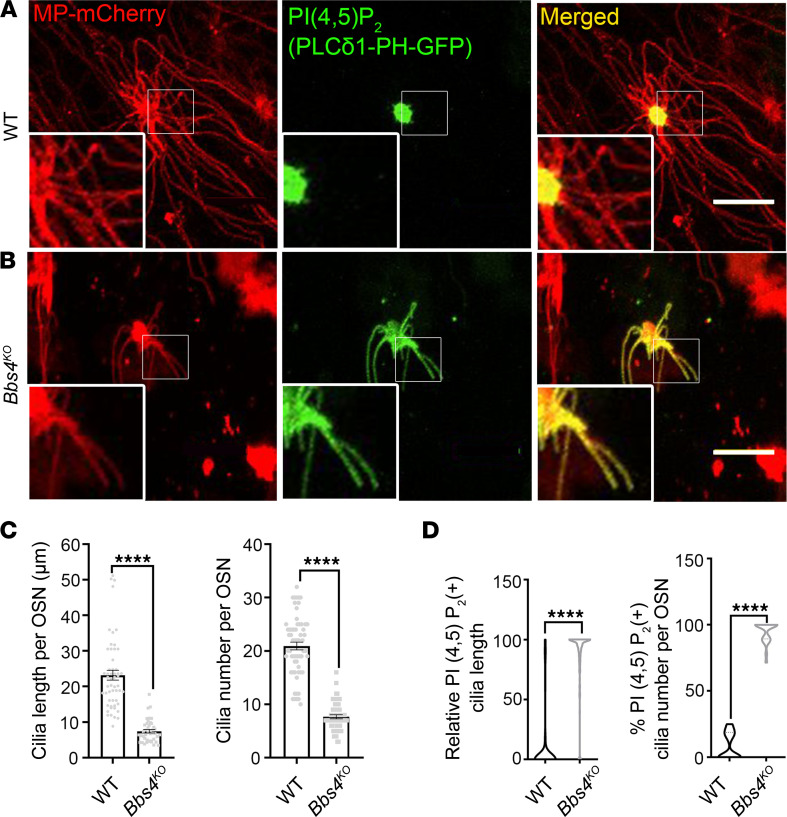
PI(4,5)P_2_ aberrantly redistributes into olfactory cilia in *Bbs4^KO^*. Representative en face images of PI(4,5)P_2_ (PLCδ1-PH-GFP) in the WT (**A**) and the *Bbs4^KO^* (**B**) olfactory cilia. At 10 days after MP-mCherry and PLCδ1-PH-GFP AV infection, the WT and *Bbs4^KO^* mice were used for en face imaging. The endogenous PI(4,5)P_2_ distribution was labeled by PLCδ1-PH-GFP and the full length of olfactory cilia was marked by MP-mCherry. Scale bars, 10 μm. (**C**) Quantification of olfactory cilia length per OSN (left) and the cilia number per OSN (right) showing *Bbs4^KO^* OSNs have significantly shorter (WT [*n* = 54 OSNs] vs. *Bbs4^KO^* [*n* = 43 OSNs]: 23.18 ± 1.382 μm vs. 7.411 ± 0.4752 μm, respectively) and fewer olfactory cilia (WT [*n* = 61 OSNs] vs. *Bbs4^KO^* [*n* = 51 OSNs]: 20.92 ± 0.7240 vs. 7.667 ± 0.3816, respectively) than WT. (**D**) Quantification of relative PI(4,5)P_2_-positive OSN cilia length (left) and the percentage of PI(4,5)P_2_-positive cilia per OSN (right) showing *Bbs4^KO^* OSNs have relatively longer (WT [*n* = 161 cilia] vs. *Bbs4^KO^* [*n* = 124 cilia]: 8.347 ± 1.950 vs. 88.98 ± 2.078, respectively) and more PI(4,5)P_2_ (WT [*n* = 23 OSNs] vs. *Bbs4^KO^* [*n* = 24 OSNs]: 8.399 ± 2.102 vs. 94.63 ± 1.649, respectively) redistributed cilia. Unpaired *t* test, ****P < 0.0001. Values represent mean ± SEM.

To understand the underlying mechanism of olfactory cilia shortening in BBS and investigate if lipid remolding occurs in olfactory cilia in vivo, we examined in a previous study the olfactory ciliary distribution of PI(4,5)P_2_ in a *Bbs4* global knockout (*Bbs4*^−/−^) mouse model (referred to as *Bbs4^KO^*) (9). WT and *Bbs4^KO^* mice were intranasally coinfected with myristoylated-palmitoylated-mCherry (MP-mCherry) and PLCδ1PH-GFP adenovirus (AV). MP-mCherry is an inert, lipid-anchored fluorophore and was used to mark the full length of olfactory cilia (9, 18). The PLCδ1-PH domain binds to PI(4,5)P_2_ with high affinity and, therefore, was applied to label the endogenous PI(4,5)P_2_ (35). En face confocal imaging was performed 10 days after the virus infection (37). Previous laboratory work showed that the mature OSNs are the only neurons in the olfactory epithelium that can be infected by AV (38). As in our previous study (9), *Bbs4^KO^* OSNs have significantly shorter and fewer olfactory cilia compared with WT OSNs (Figure 1, A and B, left panel; Figure 1C). Interestingly, PI(4,5)P_2_ olfactory ciliary distribution was significantly different between the WT and the *Bbs4^KO^* groups (Figure 1). As shown in the representative images, the distribution of PI(4,5)P_2_ was restricted to the knob of the majority OSNs in the WT mice (Figure 1A, middle panel). However, PI(4,5)P_2_ lost its restriction in the knob of OSNs and aberrantly redistributed into the residual olfactory cilia in the *Bbs4^KO^* mice (Figure 1B, middle panel). The relative PI(4,5)P_2_-positive cilia length to the full length of the cilia increased from 8.347% ± 1.950% in the WT group to 88.98% ± 2.078% in the *Bbs4^KO^* group (Figure 1D, left). The percentage of PI(4,5)P_2_-positive cilia per OSN significantly changed from 8.399% ± 2.102% in the WT group to 94.63% ± 1.649% in the *Bbs4^KO^* group (Figure 1D, right).

To further explore if PI(4,5)P_2_ ciliary mislocalization is a shared mechanism underlying olfactory dysfunction in ciliopathies, we investigated the PI(4,5)P_2_ ciliary distribution in different ciliopathies, including *Bbs1^M390R/M390R^* (homozygous for *Bbs1*M390R) and *Ift88^OSNKO^* (OSN-specific Ift88 knockout) mouse models, which all have shortened olfactory cilia (10, 14). Interestingly, similar to *Bbs4^KO^*, the *Bbs1^M390R/M390R^* group had abnormal PI(4,5)P_2_ ciliary localization (Supplemental Figure 1C; supplemental material available online with this article; https://doi.org/10.1172/jci.insight.158736DS1). However, this effect differed in *Ift88^OSNKO^* olfactory cilia, in which much shorter and fewer cilia had PI(4,5)P_2_ ciliary localization (Supplemental Figure 1, B, D, and E). Compared with the WT and *Ift88^OSNKO^* mice, the percentage of PI(4,5)P_2_-positive cilia and the relative PI(4,5)P_2_-positive cilia length were significantly increased in the *Bbs1^M390R/M390R^* and *Bbs4^KO^* mice (Supplemental Figure 1, D and E). Together, these results show that PI(4,5)P_2_ abnormally redistributes into the olfactory cilia in BBS mouse models, which indicates that PI(4,5)P_2_ ciliary redistribution is a shared mechanism for olfactory cilia shortening in BBS-mutant mice.

### PI(4,5)P_2_ is necessary for olfactory cilia shortening in Bbs4^KO^ mice.

**Figure 2:**
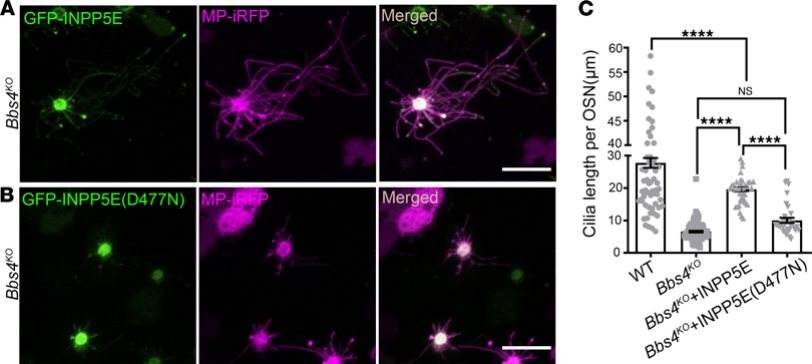
Ectopic expression of INPP5E rescues olfactory cilia length in *Bbs4^KO^*. Representative en face images of adenovirally expressed GFP-INPP5E (**A**) or GFP-INPP5E (D477N) (**B**) and MP-iRFP in olfactory cilia of *Bbs4^KO^*. Scale bars, 10 μm. (**C**) Quantification of olfactory cilia length per OSN showing a significant increase in cilia length of *Bbs4^KO^* OSNs with INPP5E infection (WT [*n* = 76 OSNs]: 27.74 ± 1.513; *Bbs4^KO^* [*n* =115 OSNs]: 6.593 ± 0.2584; *Bbs4^KO^*+INPP5E [*n* = 38 OSNs]: 19.61± 0.7611; *Bbs4^KO^*+INPP5E (D477N) [*n* = 34 OSNs]: 10.06 ± 0.7922). One-way ANOVA, ****P < 0.0001. Values represent mean ± SEM.

Next, to determine if PI(4,5)P_2_ ciliary redistribution is necessary for olfactory cilia shortening, *Bbs4^KO^* mice were adenovirally infected with MP-iRFP and GFP-INPP5E (Figure 2A), the latter of which specifically hydrolyzes PI(4,5)P_2_ in cilia (35). A catalytically inactive isoform of INPP5E, GFP-INPP5E (D477N) (35), with MP-iRFP, were administered to a different group of *Bbs4^KO^* mice and served as the negative control group (Figure 2B). The olfactory cilia length per OSN was measured 10 days after viral infection. The *Bbs4^KO^* mice receiving GFP-INPP5E (D477N) still maintained comparable olfactory cilia length per OSN to the untreated OSNs from the same animal (Figure 2C). Intriguingly, ectopic treatment with WT INPP5E, but not with INPP5E (D477N), partially rescued olfactory cilia length in *Bbs4^KO^* mice (Figure 2). Importantly, our data demonstrate that membrane remodeling of PI(4,5)P_2_ is necessary for olfactory cilia shortening in *Bbs4^KO^* mice.

### Overexpression of INPP5E rescues peripheral odor detection in Bbs4^KO^ mice.

**Figure 3:**
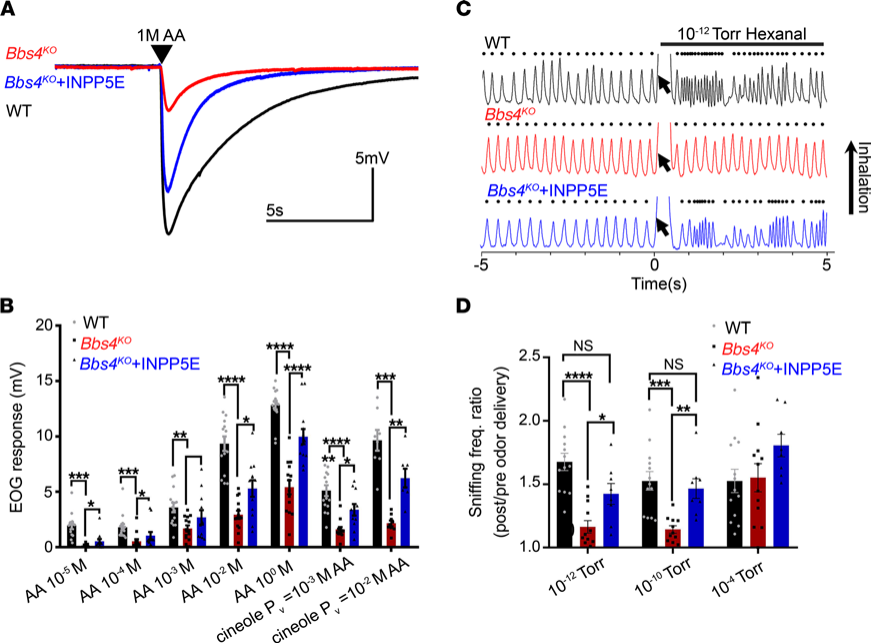
Overexpression of INPP5E rescues the impaired peripheral odor detection and odor perception of *Bbs4^KO^* mice. (**A**) Representative EOG recording traces from the surface of the olfactory epithelium of WT, *Bbs4^KO^*, and INPP5E-treated *Bbs4^KO^* (*Bbs4^KO^*+INPP5E) mice in response to the delivery of 10^0^ M AA. Arrowhead indicates the time point of odor delivery. (**B**) Quantified EOG data showing the reduced peripheral odor detection to different concentrations of AA and cineole in *Bbs4^KO^* mice had been significantly restored by ectopic expression of INPP5E. P_V_, vapor pressure. (WT, *n* = 12 animals; *Bbs4^KO^*, *n* = 10 animals; *Bbs4^KO^*+INPP5E, *n* = 10 animals.) (**C**) Representative plethysmograph traces before and during delivery of 10^–12^ Torr hexanal (arrow). Odorant (arrow) did not elicit high-frequency sniffing in *Bbs4^KO^* mice (middle), which was readily apparent in the WT (top) and INPP5E-treated *Bbs4^KO^* (*Bbs4^KO^*+INPP5E) (bottom) mice. (**D**) Detection thresholds of 13 WT, 12 *Bbs4^KO^*, and 8 *Bbs4^KO^*+INPP5E mice (average, 4 odors/mouse) indicating that reduced odorant sensitivity (i.e., increased detection thresholds) in *Bbs4^KO^* mice can be reduced by INPP5E treatment. The mouse was delivered 10 trials of vaporized mineral oil followed by presentations of an odorant at 10^–12^, 10^–10^, and 10^–4^ Torr. Sniffing frequency (freq.) ratios (sniffing Hz before vs. during odor) were compared between groups. One-way ANOVA, ****P < 0.0001, ***P < 0.001, **P < 0.01, *P < 0.05. Values represent mean ± SEM.

Defects in olfactory cilia impair the peripheral odor detection in *Bbs4^KO^* mice (9). To test whether the partial restoration of cilia length by INPP5E treatment was sufficient to restore peripheral odor detection, we performed electro-olfactogram (EOG) recording to measure the odor-evoked field potential responses on the surface of the olfactory epithelium (9, 10, 14). Compared with the untreated group, *Bbs4^KO^* mice receiving GFP-INPP5E had a significantly increased electrical response to different concentrations of amyl acetate (AA), including 10^–5^ M, 10^–4^ M, 10^–2^ M, and 10^0^ M, as well as to cineole (equal vapor pressure with 10^–3^ M AA and 10^–2^ M AA) (Figure 3, A and B). The GFP-INPP5E (D477N) treatment in *Bbs4^KO^* mice did not change their peripheral odor detection (Supplemental Figure 2). These data show that blocking of PI(4,5)P_2_ ciliary distribution by INPP5E treatment can restore the cellular odor detection in populations of peripheral olfactory neurons of *Bbs4^KO^* mice.

### Bbs4^KO^ mice have an impaired odor detection threshold, which can be rescued by treatment with INPP5E.

To further examine the therapeutic potential of INPP5E treatment in the restoration of the olfactory function, we explored the odor perception/odor detection threshold of *Bbs4^KO^* mice using whole-body plethysmography. This method takes advantage of a mouse’s innate increase in sniffing rate upon detection of a novel odorant (39) and provides a sensitive behavioral platform to quantify odor perception. As shown in the representative trace of a sniffing response to 10^–12^ Torr (1 Torr = 133.32 Pa) of hexanal (Figure 3C), the sniffing rate of the WT mouse immediately increased after the odor delivery, indicating the detection of odor. However, the sniffing rate of the *Bbs4^KO^* mouse did not change upon odor delivery at the same vapor pressure, which demonstrated a deficiency in odor perception in *Bbs4^KO^* mice (Figure 3C). Furthermore, our data showed that *Bbs4^KO^* mice did not increase their sniffing rate until 10^–6^ Torr of odor delivery (Supplemental Figure 3), indicating that *Bbs4^KO^* is a hyposmic model rather than an anosmic model. In comparison with WT mice, *Bbs4^KO^* mice had significantly higher odor detection thresholds (low odor detection sensitivity). More importantly, GFP-INPP5E–treated *Bbs4^KO^* mice had an increased sniffing rate after odor delivery of 10^–12^ Torr (Figure 3, C and D), and 10^–10^ Torr (Figure 3D). All mice in different groups had comparable sniffing responses after odorant delivery at 10^–4^ Torr (Figure 3D). Together, these data show that INPP5E treatment increases olfactory cilia length to restore whole-animal odor perception, suggesting that ectopic overexpression of INPP5E is a potential treatment for olfactory dysfunction in BBS.

### F-actin infiltrates olfactory cilia in Bbs4^KO^ mice.

**Figure 4:**
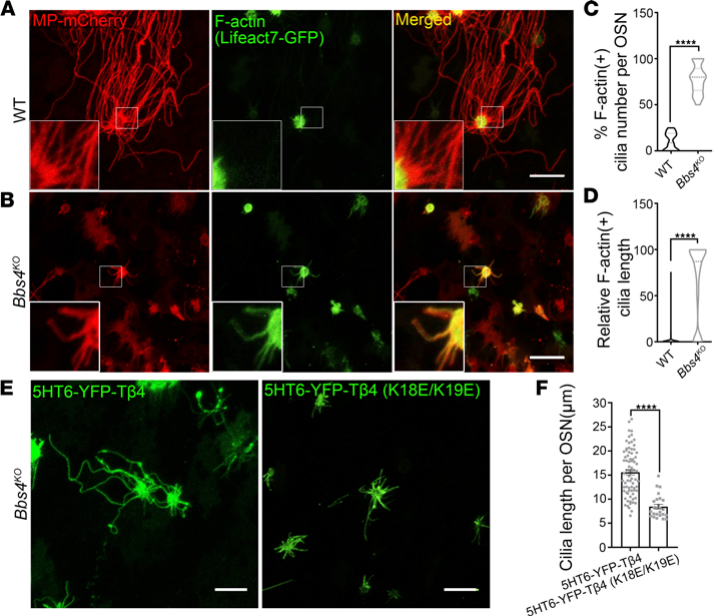
F-actin infiltrates olfactory cilia in *Bbs4^KO^*, which is necessary for *Bbs4^KO^* olfactory cilia shortening. Representative en face images of F-actin (Lifeact7-GFP) ciliary distribution in the WT (**A**) and *Bbs4^KO^* (**B**) OSNs. The WT and *Bbs4^KO^* mice were coinfected with MP-mCherry and Lifeact7-GFP AV and used for en face imaging 10 days after virus infection. MP-mCherry was used to label the full length of the olfactory cilia. Lifeact7-GFP was used to label the endogenous F-actin. (**A**) F-actin was excluded from the olfactory cilia in the WT group. (**B**) F-actin lost its restriction in the knob of OSN and was redistributed in the olfactory cilia in *Bbs4^KO^*. Scale bars, 10 μm. (**C**) Quantification data showing that the percentage of F-actin–positive cilia (F-actin–positive cilia/total cilia number × 100) was significantly increased in the *Bbs4^KO^* group (*n* = 38 OSNs; 77.32 ± 2.494) compared with the WT group (*n* = 34 OSNs; 10.37 ± 1.615). Unpaired *t* test, ****P < 0.0001. (**D**) The relative F-actin–positive cilia length (F-actin–positive cilia length/full cilia length × 100) was significantly increased in *Bbs4^KO^* (*n* = 272 cilia from 38 OSNs; 66.31 ± 2.531) compared with the WT group (*n* = 597 cilia from 34 OSNs; 1.751 ± 0.3133). Unpaired *t* test, ****P < 0.0001. (**E**) Representative en face images of the 5HT6-YFP-Tβ4–treated (left) and 5HT6-YFP-Tβ4 (K18E/K19E)–treated (i.e., the actin-binding mutant) (right) *Bbs4^KO^* olfactory cilia. Scale bars, 10 μm. (**F**) Quantification of olfactory cilia length showed that *Bbs4^KO^* olfactory cilia length was partially rescued by 5HT6-YFP-Tβ4 treatment (5HT6-YFP-Tβ4 [*n* = 51 OSNs] vs. 5HT6-YFP-Tβ4 (K18E/K19E) [*n* = 25 OSNs]: 15.60 ± 0.5596 vs. 8.448 ± 0.4769, respectively). Unpaired *t* test, ****P < 0.0001. Values represent mean ± SEM.

Next, we explored how membrane lipid remodeling induced olfactory cilia disassembly in BBS. PI(4,5)P_2_ is an important regulator of actin cytoskeletal dynamics in cells (40, 41). Elevated levels of PI(4,5)P_2_ regulate the activities of several F-actin–regulatory proteins and, therefore, promotes the polymerization of F-actin in cells (41). Recently, PI(4,5)P_2_ was shown to induce intraciliary polymerization of F-actin (27), which has emerged as a major factor in the disassembly of primary cilia in vitro (15, 27, 42, 43). To understand if PI(4,5)P_2_ olfactory ciliary remodeling induces F-actin ciliary infiltration, we measured F-actin olfactory ciliary localization in the *Bbs4^KO^* mouse model. WT and *Bbs4^KO^* mice were intranasally coinfected with MP-mCherry and Lifeact7-GFP AV to label the full length of olfactory cilia and the endogenous F-actin, respectively (9, 44). Interestingly, our data revealed that F-actin olfactory ciliary distribution was significantly different between the WT and the *Bbs4^KO^* groups (Figure 4, A–D). For the majority of OSNs in the WT group, F-actin localized in the knob of OSNs and was excluded from the olfactory cilia (Figure 4A). This was consistent with the current understanding that F-actin is excluded from the cilia structure (45). However, F-actin lost its restriction in the knob of OSNs and aberrantly infiltrated olfactory cilia in *Bbs4^KO^* mice (Figure 4B). The analysis of the data showed that the percentage of F-actin–positive cilia per OSN significantly increased from 10.37% ± 1.615% in the WT group to 77.32% ± 2.494% in the *Bbs4^KO^* group (Figure 4C). The relative percentages of F-actin–positive cilia length to the full length of the cilia significantly increased from 1.751% ± 0.3133% in the WT group to 66.31% ± 2.531% in the *Bbs4^KO^* group (Figure 4D).

To compare the F-actin ciliary localization in different ciliopathy mouse models with shortened olfactory cilia, we investigated its distribution in both *Ift88^OSNKO^* and *Bbs1^M390R/M390R^* (10, 14). Similar to PI(4,5)P_2_, F-actin did not show abundant ciliary redistribution in *Ift88^OSNKO^* olfactory cilia (Supplemental Figure 4B), but F-actin aberrantly infiltrated i*Bbs1^M390R/M390R^* olfactory cilia (Supplemental Figure 4C). Compared with the WT and *Ift88^OSNKO^* mice, the percentage of F-actin–positive cilia and the relative F-actin–positive cilia length were significantly increased in the *Bbs1^M390R/M390R^* mice (Supplemental Figure 4, D and E). Together, these results show that F-actin abnormally infiltrates the olfactory cilia in BBS mouse models but not in at least 1 other ciliopathy mouse model, indicating that the ciliary redistribution of F-actin is a shared mechanism for olfactory cilia shortening in BBS-mutant mice.

### F-actin is necessary for olfactory cilia shortening in Bbs4^KO^ mice.

We further evaluated the necessity of F-actin ciliary redistribution for olfactory cilia shortening in *Bbs4^KO^* by targeted overexpression of thymosin-β4 (Tβ4), which sequesters G-actin from incorporation into actin filaments and, therefore, regulates actin polymerization (46). Tβ4 was fused to a ciliary localized GPCR, 5HT6, which efficiently targeted Tβ4 into olfactory cilia to specifically suppress intraciliary F-actin (Supplemental Figure 5) (27). The *Bbs4^KO^* mice were divided into 2 groups and were infected with AV containing 5HT6-YFP-Tβ4 or 5HT6-YFP-Tβ4 (K18E/K19E). The 5HT6-YFP-Tβ4 (K18E/K19E) is an actin binding–deficient mutant (27) and therefore was the negative control. As expected, the adenoviral treatment with 5HT6-YFP-Tβ4 (K18E/K19E) did not affect olfactory cilia length in *Bbs4^KO^* mice (Figure 4F), which still had comparable olfactory cilia length per OSN to the untreated OSNs from *Bbs4^KO^* mice (Figure 2C). Importantly, the expression of 5HT6-YFP-Tβ4 in *Bbs4^KO^* significantly increased the olfactory cilia length per OSN (Figure 4, E and F), which was relatively shorter than that in WT group (Figure 2C), suggesting that 5HT6-YFP-Tβ4 partially rescued *Bbs4^KO^* olfactory cilia length. These data show that F-actin ciliary redistribution is necessary for olfactory cilia shortening in *Bbs4^KO^* mice.

### PI(4,5)P_2_ regulates F-actin olfactory ciliary distribution in Bbs4^KO^ mice.

**Figure 5:**
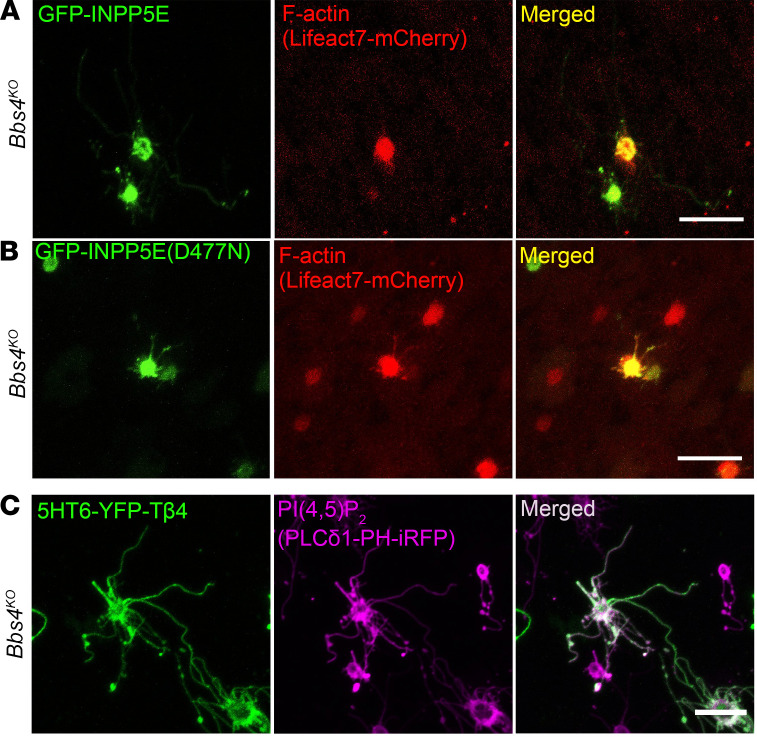
PI(4,5)P_2_ directly controls F-actin ciliary distribution in *Bbs4^KO^* OSNs. (**A**) Representative en face images of F-actin (Lifeact7-mCherry) ciliary distribution in GFP-INPP5E–infected *Bbs4^KO^* OSNs. The expression of GFP-INPP5E blocked F-actin ciliary abnormal localization in *Bbs4^KO^*. (**B**) Representative en face images of F-actin (Lifeact7-mCherry) ciliary distribution in GFP-INPP5E (D477N)–treated *Bbs4^KO^* OSNs. F-actin infiltrated the olfactory cilia after treatment with GFP-INPP5E (D477N). (**C**) En face images of PI(4,5)P_2_ (PLCδ1-PH-iRFP) ciliary redistribution in 5HT6-YFP-Tβ4–expressed *Bbs4^KO^* OSNs. PI(4,5)P_2_ still redistributed into the olfactory cilia in 5HT6-YFP-Tβ4–treated *Bbs4^KO^* OSNs. Scale bars, 10 μm.

To understand if PI(4,5)P_2_ is involved in the regulation of actin polymerization in olfactory cilia, we investigated the interrelationship between PI(4,5)P_2_ and F-actin in olfactory cilia. Our data showed that F-actin exclusion from cilia was reestablished by blocking PI(4,5)P_2_ ciliary redistribution in GFP-INPP5E–treated *Bbs4^KO^* mice (Figure 5A). As expected, F-actin still redistributed into the olfactory cilia in the GFP-INPP5E (D477N)–treated *Bbs4^KO^* group (Figure 5B), in which PI(4,5)P_2_ localized to cilia. On the contrary, blocking ciliary F-actin by 5HT6-YFP-Tβ4 did not prevent PI(4,5)P_2_ ciliary redistribution in *Bbs4^KO^* mice (Figure 5C). Together, these results show that ciliary-localized PI(4,5)P_2_ directly regulates F-actin olfactory ciliary redistribution in *Bbs4^KO^* mice.

### Bbs4 single-gene replacement restores olfactory ciliary exclusion of F-actin and PI(4,5)P_2_ in Bbs4^KO^ mice.

It has been demonstrated that intranasal adenoviral and adeno-associated virus–mediated gene delivery of WT genes can restore ciliary morphology and olfactory function in ciliopathy mouse models (9, 10, 14). The olfactory cilia shortening in *Bbs4^KO^* mice can be partially rescued by *Bbs4* gene replacement (9). To investigate the underlying mechanism of this ciliary length rescue, we explored the ciliary distribution of F-actin and PI(4,5)P_2_ after *Bbs4* single-gene replacement. Compared with the untreated group, OSNs in *Bbs4^KO^* mice with the expression of BBS4-mCherry showed no F-actin ciliary localization (Supplemental Figure 6A). Furthermore, normal ciliary distribution of PI(4,5)P_2_ in OSNs was restored in *Bbs4^KO^* mice after *Bbs4* gene replacement (Supplemental Figure 6B). Overall, these data suggest that *Bbs4* gene replacement rescues olfactory cilia length in *Bbs4^KO^* mice by excluding abnormally distributed F-actin and PI(4,5)P_2_ in olfactory cilia.

## Discussion

**Figure 6:**
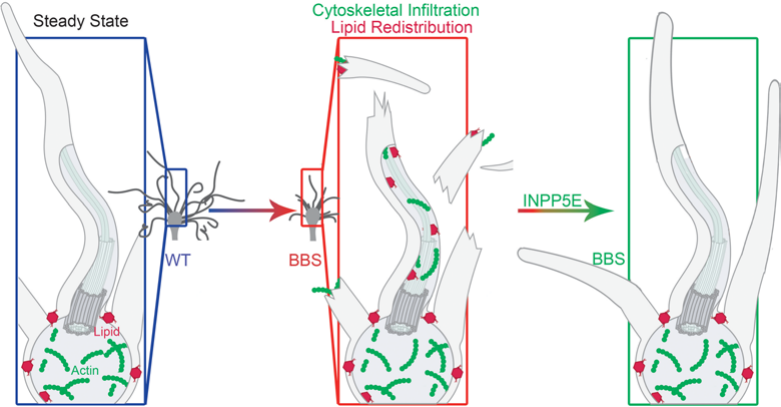
Schematic representation of the underlying mechanisms of olfactory cilia shortening in BBS. The distributions of PI(4,5)P_2_ and F-actin are normally restricted to the knob of the WT OSN. However, in olfactory cilia in BBS, the dysfunctions in the BBSome lead to aberrant PI(4,5)P_2_ ciliary redistribution and F-actin infiltration, which are necessary for olfactory cilia shortening and contribute to the pathogenesis of BBS. Blocking PI(4,5)P_2_ and F-actin ciliary mislocalization by adenoviral expression of INPP5E restores olfactory cilia length in BBS, which, therefore, rescues defects in peripheral odor detection and odor perception of BBS.

Our work demonstrates that aberrant ciliary redistribution of PI(4,5)P_2_ and F-actin are necessary for olfactory cilia disassembly and contribute to the pathogenesis of BBS (Figure 6). More importantly, blocking PI(4,5)P_2_ and F-actin ciliary mislocalization by adenoviral expression of INPP5E restores olfactory cilia length in *Bbs4^KO^* mice (Figure 6), which is sufficient to rescue peripheral odor detection and reestablish odor perception at the whole-animal level. This study provides valuable insights into mechanisms of olfactory cilia disassembly in pathological conditions and highlights viable candidate targets for the treatment of olfactory dysfunction and other symptoms of ciliopathies.

Olfactory cilia have unique membrane-lipid compositions (35) due to the presence of the transition zone (TZ), which strictly controls the localization of ciliary protein and membrane lipids (32, 47–49). Ciliary phosphoinositides are emerging as critical regulators in primary cilia (50); however, their role in the biogenesis and maintenance of olfactory cilia is poorly understood. PI(4,5)P_2_ is a phosphoinositide that is restricted to the membrane of the ciliary base and is absent from cilia as a result of its hydrolyzation by INPP5E (27, 32–35). Intriguingly, our in vivo work revealed for the first time, to our knowledge, that not only is PI(4,5)P_2_ aberrantly redistributed into cilia in terminally differentiated neurons of BBS mice (Figure 1 and Supplemental Figure 1) but also is necessary for olfactory cilia shortening (Figure 2). This finding is supported by studies of primary cilia in which the ciliary remodeling of PI(4,5)P_2_ caused cilia disassembly or ciliary fission in cells entering the cell cycle, as well as those under normal and agonist stimulation conditions (27, 51). Although different in that cell cycle–mediated cilia disassembly is a normal physiological process, our study in nondividing neurons in pathological conditions shows a similar mechanism involved in the loss of cilia. This suggests that the aberrant ciliary redistribution of PI(4,5)P_2_ is a conserved mechanism involved in the disassembly of cilia. Additionally, there is controversy regarding the sufficiency of PI(4,5)P_2_ ciliary redistribution for cilia shortening, because its accumulation had opposing effects on the primary cilia of 2 distinct cell types (27, 49, 51). Interestingly, our previous study showed that PI(4,5)P_2_ ciliary redistribution through deletion of INPP5E in OSNs did not reduce olfactory ciliary length (35). Together, the findings from our studies suggest that the ciliary mislocalization of PI(4,5)P_2_ is necessary but not sufficient for olfactory cilia shortening and highlight that the role of PI(4,5)P_2_ in the maintenance of cilia is cell type specific.

In addition to PI(4,5)P_2_, the role of other ciliary phosphoinositides in olfactory cilia is largely underexplored. For instance, our previous work showed that phosphatidylinositol-3,4,5-trisphosphate [PI(3,4,5)P_3_] was restricted mostly to the knobs of OSNs, with relatively low presence in olfactory cilia (35). Depletion of phosphatase and tensin homolog, an enzyme that converts PI(3,4,5)P_3_ to PI(4,5)P_2_ (52), promotes primary cilia disassembly (53), suggesting that PI(3,4,5)P_3_ may be involved in the process of cilia disassembly. Additional efforts should be made to explore the role of other ciliary phosphoinositides in regulating the dynamics of olfactory cilia.

As microtubule-based organelles, olfactory cilia, like other types of cilia, were believed to not contain F-actin. However, emerging work in cultured cells has shown that primary cilia disassembly can occur through F-actin–dependent mechanisms in both agonist and growth stimulation conditions (26, 27, 42). Our observations showed that in BBS F-actin abnormally infiltrated olfactory cilia (Figure 4, A–D). Importantly, overexpression of the actin-sequestering protein Tβ4 significantly rescued olfactory cilia length in BBS (Figure 4, E and F), providing strong evidence that the loss of olfactory cilia in BBS is mediated by F-actin–dependent cilia disassembly. This raises a question of whether F-actin–dependent cilia disassembly is a conserved mechanism for cilia loss in other ciliopathies. Interestingly, treatment with cytochalasin D has been implicated in restoring the loss of primary cilia in cultured cells with the IFT88 hypomorphic mutation (orpk/orpk) (54), suggesting that F-actin contributed to the loss of primary cilia in the IFT88-mutant model. In contrast, our results showed that F-actin displayed a different olfactory ciliary distribution pattern in *Ift88^OSNKO^* than in BBS (Supplemental Figure 4, B, D, and E). The seemingly contradictory findings may be explained by differences between cilia types and working models. Nevertheless, our work suggests that the loss of olfactory cilia in *Ift88^OSNKO^* is not caused by F-actin–mediated cilia disassembly but likely induced by dysfunction of cilia assembly due to the disruption of IFT. Regardless, F-actin emerges as a candidate target in the treatment of olfactory cilia dysfunction induced by cilia disassembly.

An important factor to understand the mechanisms of olfactory cilia disassembly is the timing of each step. Our results showed that the ciliary infiltration of F-actin was induced by the ciliary redistribution of PI(4,5)P_2_ (Figure 5) and are supported by results in primary cilia (27, 51). The results provide further insight into how membrane remolding induces olfactory cilia disassembly in BBS. Within cells, PI(4,5)P_2_ is an important regulator of actin dynamics through interactions with actin-regulatory proteins (55). In fact, in vitro studies show that the loss of actin regulators that facilitate the polymerization of actin significantly increased primary cilia length (54, 56, 57). This indicates that actin regulators may be highly involved in the shortening of olfactory cilia in BBS. Therefore, additional work is necessary to understand which and how actin regulators may participate in olfactory cilia shortening in BBS.

BBS is a highly pleiotropic disease associated with variable penetrance and phenotypes within different organ systems (8, 58). The deletion or mutation of BBS proteins markedly decreases cilia length and number in OSNs (9, 10) but not in several other ciliated systems, including the brain (20, 59), the respiratory system (9, 24, 60), and kidney cells (61), suggesting that BBSome functions in the maintenance of olfactory cilia may be unique (9, 21). The results showing that PI(4,5)P_2_ and/or F-actin abnormally localize to olfactory cilia in multiple BBS models, but not in *Ift88^OSNKO^* mice (Supplemental Figures 1 and 4), may reveal a novel role of the BBSome in OSNs. The ectopic expression of WT BBS4 prevented PI(4,5)P_2_ and F-actin ciliary mislocalization, suggesting that the BBSome may be required for the proper ciliary localization of phospholipid and actin filaments. These concepts are supported by evidence from other cilia types (16, 62, 63). Our work provides direct evidence that the BBSome plays a significant role in regulating the integrity of olfactory cilia, including the control of membrane-lipid composition, which is critical for the maintenance of proper cilia length and function.

There are several intriguing yet unanswered questions, one of which is how PI(4,5)P_2_ and F-actin aberrantly redistribute into olfactory cilia in BBS. Clues for potential underlying mechanisms may reside in studies in other cilia types. Evidence from zebrafish (64) and mice (36, 48) shows that the BBSome interacts with the TZ and has overlapping roles in regulating primary ciliogenesis (65). A study in human renal tubular cells shows that INPP5E was absent from primary cilia with a dysfunctional TZ (66). Importantly, deletion of BBS4 in mouse embryonic fibroblasts also results in a significantly reduced ciliary localization of INPP5E (36). Together, these pieces of evidence highlight a possible underlying mechanism whereby dysfunction of the BBSome causes defects in the TZ, in turn decreasing the level of INPP5E, thus inducing the ciliary accumulation of PI(4,5)P_2_ and F-actin. Another question is, how do PI(4,5)P_2_ and/or F-actin ciliary mislocalization lead to shorter olfactory cilia? A potential mechanism is that the aberrant ciliary infiltration of F-actin disrupts the stability of the ciliary microtubule structure and causes cilia disassembly. Supporting evidence for this hypothesis is presented in a study of Xenopus egg extracts, showing that branched F-actin generates a mechanical force that blocks microtubule growth and triggers the disassembly of microtubule structures (67). Alternatively, membrane tension plays crucial roles in regulating the dynamics of membrane and cellular processes. A high concentration of PI(4,5)P_2_ or F-actin in cells can result in an increase in membrane tension (68–70). Failure to maintain normal membrane tension can lead to membrane lysis (68, 70). Based on these findings, we hypothesize that ciliary mislocalized PI(4,5)P_2_ and F-actin alter the ciliary membrane tension and impair the dynamic of the ciliary membrane, which, in turn, result in cilia shortening. These potential mechanisms describing the pathogenesis of BBS need to be examined.

Previous laboratory work demonstrated that the loss of BBS4 in mice causes defects in peripheral odor detection, by measurement of EOG recordings (9). However, it remained unknown if loss of BBS4 induced odor perception defects at the whole-animal level. Our work using whole-body plethysmography showed that *Bbs4^KO^* mice had a higher odor detection threshold, meaning lower odor detection sensitivity, compared with WT mice. Importantly, *Bbs4^KO^* mice showed a similar response to that of WT mice after odor delivery of 10^−6^ Torr and higher vapor pressures. This result shows that *Bbs4^KO^* mice have a shift in odor detection threshold instead of a complete loss of odor detection, due to the shortened and loss of olfactory cilia. Our work shows that the *Bbs4^KO^* mouse model is a hyposmic model rather than an anosmic model, which is consistent with clinical findings in patients with BBS (24). That the sniffing curves of *Bbs4^KO^* and WT mice were similar at higher concentrations suggests that suprathreshold magnitude is unchanged in the *Bbs4^KO^* mice. This highlights the tremendous spare capacity of the olfactory system, which likely helps maintain the integrity of the neural circuitry necessary for odor perception. Furthermore, the partial recovery of olfactory cilia length by the overexpression of INPP5E in *Bbs4^KO^* mice rescued the odor detection threshold, suggesting the restoration of olfactory input is necessary for the treatment of olfactory dysfunction. Together, these observations demonstrate the potential that olfactory dysfunction could be fully rescued in patients with BBS.

Our work highlights the potential of using common mechanisms in olfactory cilia shortening as therapeutic targets for the treatment of olfactory dysfunction in BBS and other ciliopathies. Gene therapy is a promising curative approach for olfactory dysfunction in ciliopathies (9, 10, 14, 15). Single-gene replacement mediated by intranasal AV and/or adeno-associated viruses was capable of rescuing the morphology and odor detection of olfactory cilia in ciliopathy mouse models (9, 10, 14). However, the single-gene replacement approach is limited, because it only can be applied to a subset of patients with dysfunction in the corresponding gene (15). Rather than using WT BBS4 gene replacement to rescue *Bbs4^KO^* olfactory cilia, we restored the cilia length in this study by reversing the aberrant distribution of PI(4,5)P_2_ and F-actin, 2 common factors that contribute to the pathogenesis of multiple BBS (Figure 2 and Figure 4, E and F). Strikingly, the recovery of olfactory cilia length by INPP5E treatment was sufficient to restore peripheral odor detection and even rescue the odor perception in *Bbs4^KO^* mice (Figure 3), suggesting that INPP5E is a potential treatment for olfactory dysfunction in BBS. Beyond olfactory impairment, BBS is highly pleiotropic; thus, future studies should investigate if a similar mechanism causes cilia disassembly and if INPP5E treatment could restore the morphology and function of cilia in other ciliated organ systems. Furthermore, it is possible that PI(4,5)P_2_- and F-actin–mediated cilia disassembly not only is involved in cilia loss in BBS but also contributes to the pathogenesis of other ciliopathies. Overall, our work highlights the potential of INPP5E as a pan-treatment for ciliopathies, thus moving beyond single-gene replacement and benefitting a broader patient population.

## Methods

### Mice.

The male and female mice were bred and maintained at the University of Florida. The *Bbs4^KO^*, *Bbs1^M390R/M390R^*, and *IFT88^OSNKO^* mice and their WT littermates of both sexes were used for experiments. Genotyping was performed according to previously published work (9, 14, 20).

### Plasmids and AV production.

AVs MP-GFP, MP-mCherry, MP-iRFP, BBS4-mCherry, PLCδ1-PH–GFP, GFP-INPP5E, and GFP-INPP5E-D477N were validated and described previously (9, 10, 35). Plasmids containing cDNA fragments were provided as follows: Lifeact7-GFP (Addgene plasmid 54610) were deposited by Michael Davidson (National High Magnetic Field Laboratory, Florida State University, Tallahassee, Florida, USA); 5HT6-YFP-Tβ4 (Addgene plasmid 96806) and 5HT6-YFP-Tβ4 (K18E/K19E) mutant (Addgene plasmid 96807) were deposited by Takanari Inoue (Johns Hopkins University School of Medicine, Baltimore, Maryland, USA) (27). As previously described (9), all cDNAs were fused with fluorescence expression sequence and inserted into the pAd/CMV/V5-DEST expression vector using Gateway technology (Invitrogen). AV was produced and amplified in HEK293 cells (ATCC) using the ViraPower protocol (Invitrogen). The Adenovirus Mini Purification Virakit (Virapur) was used for the isolation and purification of AV. The dialysis of the virus was performed in 2.5% glycerol, 25 mM NaCl, and 20 mM Tris-HCl (pH 8.0) using a 10,000 MWCO Slide-A-Lyzer dialysis cassette (Thermo Fisher Scientific) at 4°C overnight. Then AV was aliquoted and stored at −80°C for experimentation.

### Intranasal viral administration.

AV coding for fluorescence-tagged target proteins was intranasally administered to mice at P7, P21, or 3–4 months of age, as previously described (9). To better perform the viral infection, mice at P7 and P21 were restricted by hand, and mice at 3–4 months were anesthetized with ketamine/xylazine. Using a pulled 1 mL syringe, the virus was administered by applying a series of small drops to the nasal cavity of mice. The intranasal delivery of the virus was alternated between the right and left nostrils to avoid potential drowning. At 10 days after the 3 subsequent days of the viral infection, the mice were used for experiments.

### Live en face confocal imaging.

The AV-infected animals (age P21) were euthanized with CO_2_, and then the olfactory turbinates were exposed as previous described (9). The tissue was placed with the turbinate surface facing down in a bath of freshly oxygenated, artificial cerebrospinal fluid (124 mM NaCl, 3 mM KCL, 1 mM MgCl_2_, 2 mM CaCl_2_, 1.25 mM NaH_2_PO_4_, 26 mM NaHCO_3_, 25 mM glucose) and was gently held down using a mesh within the chamber. The imaging was performed on a Nikon TiE-PFS-A1R confocal microscope. The images and cilia length measurements were processed and performed using Fiji–ImageJ software (NIH). Final figures were assembled using Photoshop 6CS (Adobe).

### EOG recording.

After euthanizing mice with CO_2_, their olfactory turbinates (at the age of P30–P35) were exposed for EOG, which was recorded from multiple turbinates using a MultiClamp 700A amplifier controlled by pClamp software (Molecular Devices). Electrodes were made from standard glass micropipettes filled with 0.5% SeaPlaque agarose (Sigma-Aldrich) in 1× PBS. All odorants, including AA and cineole (Sigma-Aldrich), were diluted in DMSO (Sigma-Aldrich) and mixed to the final working concentration (as shown in Figure 3B) in ultrapure water. Then odorants were delivered in vapor phase along with the humidified airflow to the surface of the tissue. Tissues were allowed 1 minute between subsequent odor deliveries to reduce the adaptation of the EOG response to the previous odorant. The data were analyzed with Clampfit (Molecular Devices).

### Whole-body plethysmography.

To avoid the potential artifact induced by the motivation deficits in global ciliopathy mouse models, whole-body plethysmography was used to determine odor detection thresholds. The assay takes advantage of the innate behavior that mice have of increasing their sniffing rate when presented with a novel stimulus (39, 71). The whole-body plethysmography was controlled by the pClamp software (Molecular Devices). Mice at 3–4 months old were used for this test. Four odorants — hexanal, cineole, propionic acid, and AA (Sigma-Aldrich) — were used in the experiment. All odorants were diluted in mineral oil (Sigma-Aldrich) in log series and delivered in the vapor phase with constant air delivery (1 L/min) into the plethysmograph chamber. To avoid the potential artifact induced by the background odor, the chamber, and the possible pressure changes, which may be associated with the odor delivery, animals were habituated to the experimental setting 20 min/d with 10× mineral oil–vapor delivery within the chamber, for 3 days before the experiment. The recordings were done on 4 consecutive days, each of which contained 10 trials of mineral oil followed by the delivery of an odorant at 10^–12^, 10^–10^, 10^–8^, 10^–7^, 10^–6^, 10^–5^, 10^–4^, 10^–3^, 10^–2^, and 10^–1^ Torr. The odor detection threshold data were collected from the response of each mouse to 4 different odorants. Sniffing frequency ratios (sniffing rate 5 seconds before vs. 5 seconds after odor delivery) were calculated with Clampfit and compared between groups.

### Statistics.

All values in reported in Results are presented as mean ± SEM. The graph making and statistical analysis were performed with Prism 8 software (GraphPad Software). The *t* test (2-tailed) was used for comparison of the results between 2 groups and 1-way ANOVA was used to calculate the statistical significance among multiple groups. *P* < 0.05 was considered significant.

### Study approval.

All procedures involving animals in this study were approved by the University of Florida IACUC.

## Author contributions

CX and JRM designed the research experiments. CX, JCH, KU, and RJC performed the experiments. CX, KU, CRU, and LZ generated reagents. CX generated the figures and analyzed the data. CX and JRM wrote the manuscript, with JCH providing critical input. All authors participated in revising the final manuscript and approved the final version.

## Supplementary Material

Supplemental data

## Version 1 06/30/2022

In-Press Preview

## Version 2 08/08/2022

Electronic publication
