# Supplementary material for: Reversal of ciliary mechanisms of disassembly rescues olfactory dysfunction in ciliopathies
Source: JCI Insight. 2022 Aug 8;7(15):e158736. doi: 10.1172/jci.insight.158736 (PMC9462494; doi:10.1172/jci.insight.158736)
Supplement: Supplemental data [file jciinsight-7-158736-s073.pdf]

Supplemental Material.

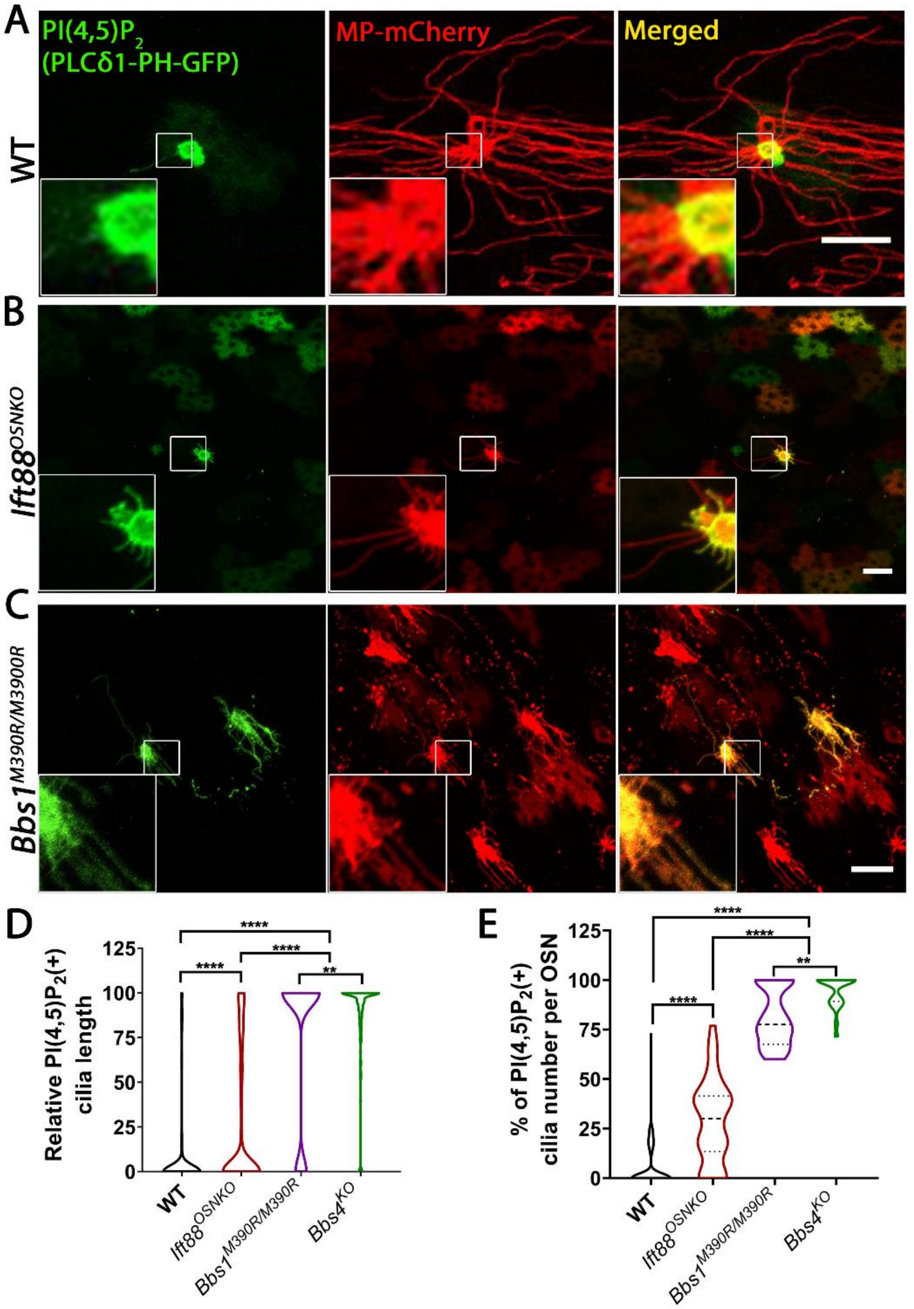

Supplementary Figure 1. $PI(4,5)P_2$ ciliary localization in other ciliopathy models. Representative *en face* images of $PI(4,5)P_2$ (PLCδ1-PH-GFP) in the WT **(A)**, *$Ift88^{OSNKO}$***(B)**, and *$Bbs1^{M390R/M390R}$* **(C)** olfactory cilia. The mice were infected with PLCδ1PH-GFP and MP-mCherry adenovirus and used for *en face* imaging. MP-mCherry was used to label the full length of the olfactory cilia. PLCδ1-PH-GFP was used to label the endogenous $PI(4,5)P_2$. Scale 10 μm. **(D)** Quantification data showing that the relative $PI(4,5)P_2$ positive cilia length ($PI(4,5)P_2$ positive cilia length/ full cilia length*100) was increased in the BBS group compared to the WT and *$Ift88^{OSNKO}$* group, One way ANOVA, ****$p<0.0001$. **(E)** The percentage of $PI(4,5)P_2$ positive cilia number ($PI(4,5)P_2$ positive cilia number/total cilia number*100) was increased in the BBS group compared to the WT and *$Ift88^{OSNKO}$* group. One way ANOVA, ****$p<0.0001$. Values represent means ± SEM.

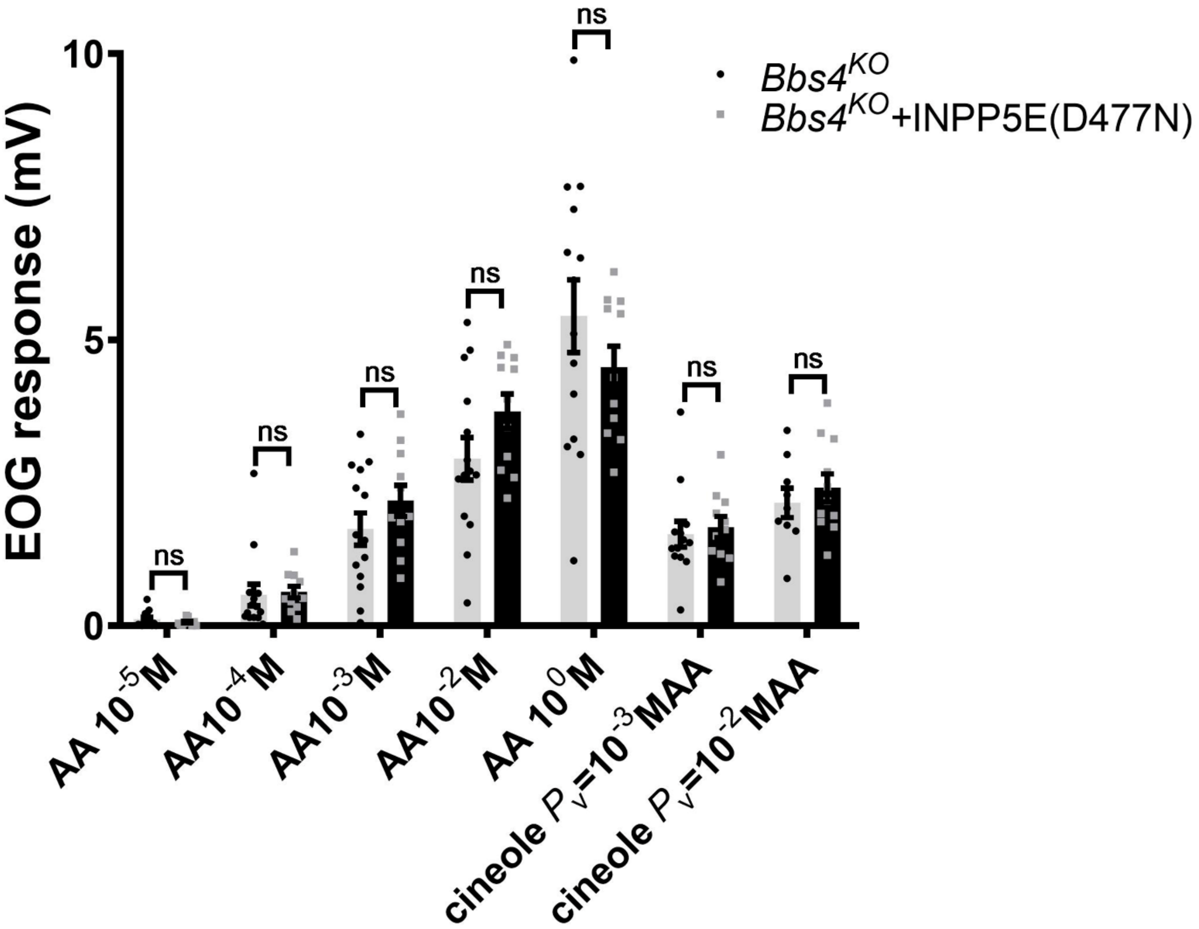

Supplementary Figure 2. Ectopic expression of INPP5E(D477N) does not rescue the impaired peripheral odor detection in *Bbs4*^{KO} mice. Quantified EOG data of 1month *Bbs4*^{KO} mice and AV-GFP-INPP5E(D477N) treated *Bbs4*^{KO} mice in response to different concentrations of amyl acetate (AA), and cineole. EOG response was not restored by ectopic expression of INPP5E(D477N). PV: vapor pressure. (*Bbs4*^{KO}: n=10 animals; *Bbs4*^{KO}+INPP5E(D477N): n=11 animals). Multiple *t-test*, ns: non-significant. Values represent means ± SEM.

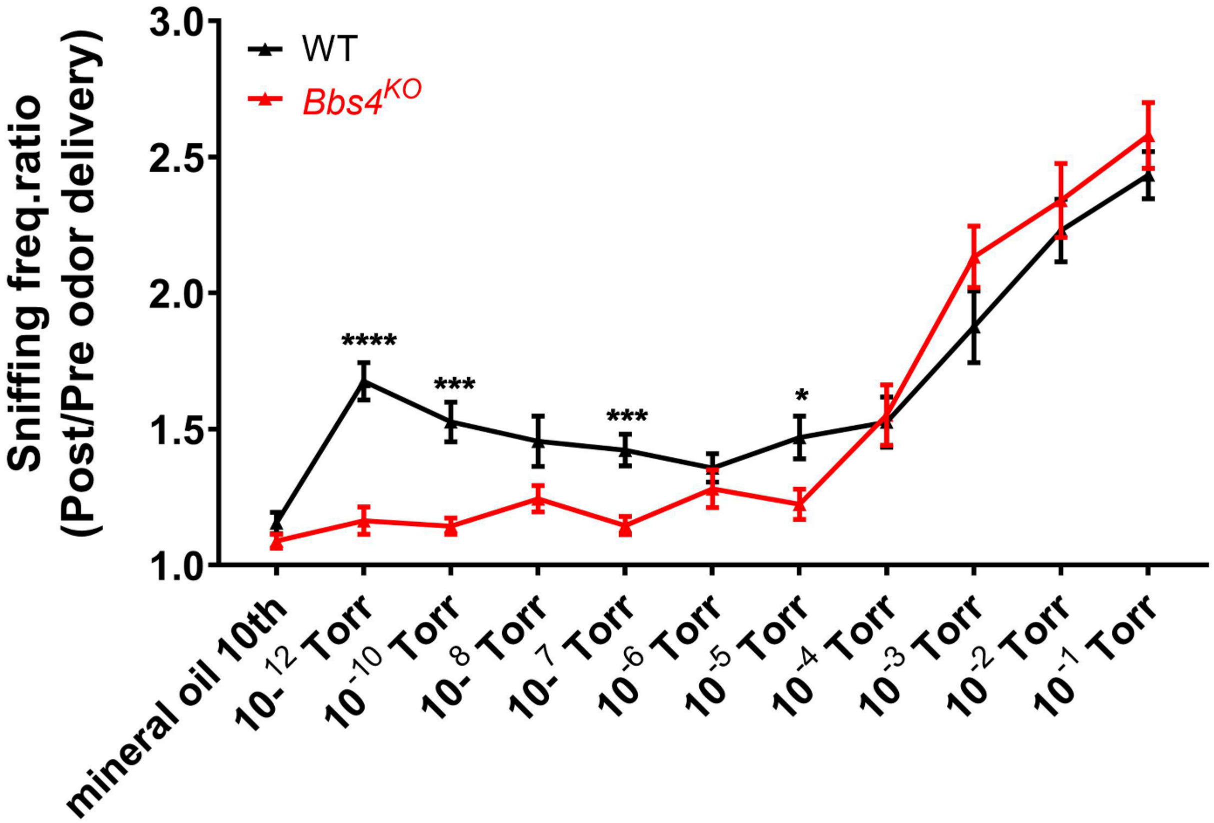

Supplementary Figure 3. *Bbs4*^{KO} mice exhibit a higher odor detection threshold. Odor detection thresholds of WT mice (n=13) and *Bbs4*^{KO} mice (n=12) (average of 4 odors / mouse) from an ascending staircase paradigm indicating that *Bbs4*^{KO} mice had reduced odorant sensitivity (increased detection thresholds). Each mouse was delivered 10 trials of vaporized mineral oil followed by presentations of an odorant at 10^{-12}, 10^{-10}, 10^{-8}, 10^{-7}, 10^{-6}, 10^{-5}, 10^{-4},10^{-3}, 10^{-2}, and 10^{-1}Torr. Sniffing frequency ratios (sniffing Hz pre vs during odor) were compared between groups. One way ANOVA, ****$p < 0.0001$. ***$p < 0.001$. **$p < 0.01$.*$p < 0.05$. Values represent means ± SEM.

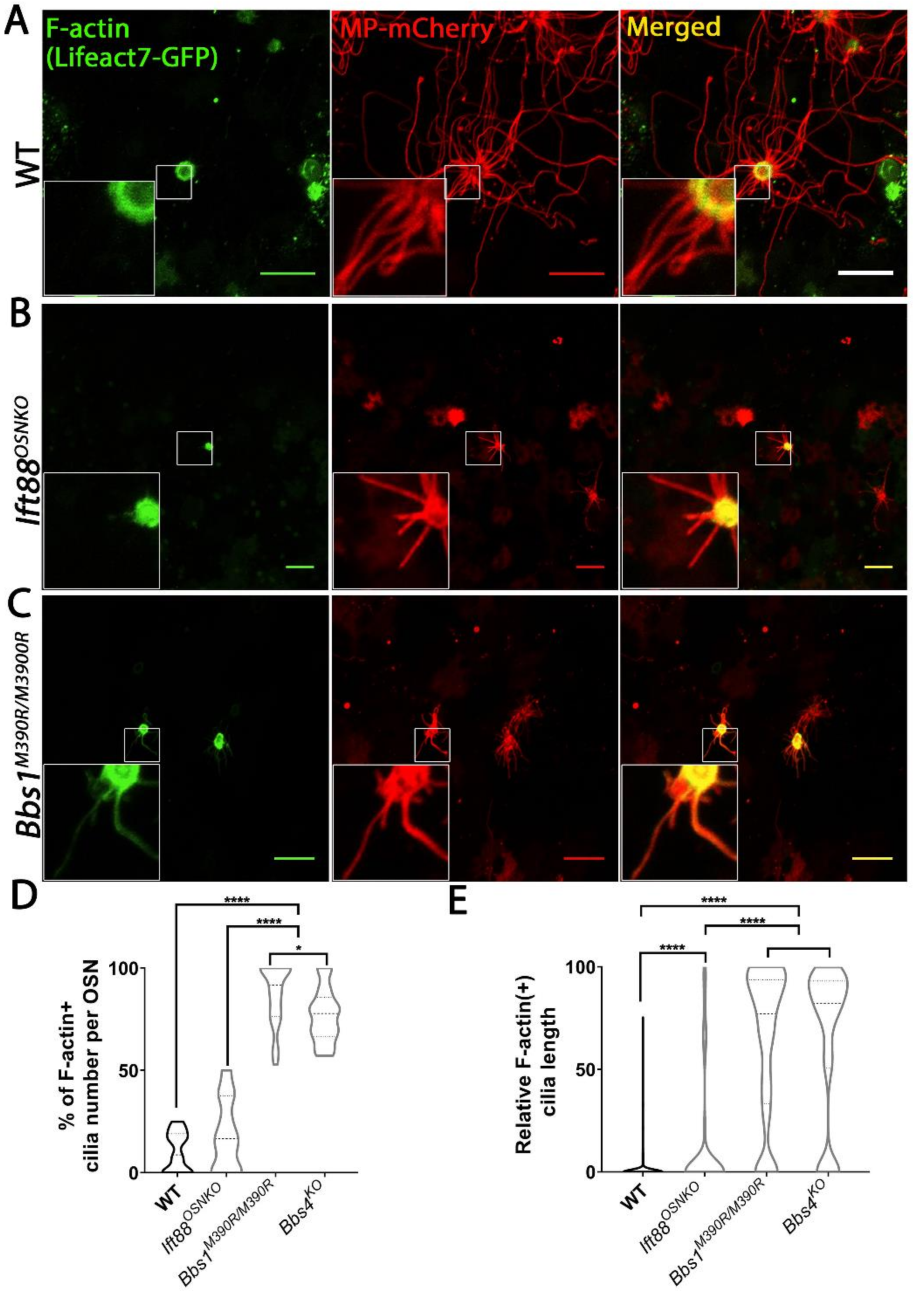

Supplementary Figure 4. F-actin ciliary localization in other ciliopathy models. Representative en face images of F-actin (Lifeact7-GFP) in the WT **(A)**, *Ift88*^{OSNKO} **(B)**, and *Bbs1*$^{M390R/M390R}$ **(C)** olfactory cilia. The mice were infected with MP-mCherry and Lifeact7-GFP adenovirus and used for en face imaging. MP-mCherry was used to label the full length of the olfactory cilia. Lifeact7-GFP was used to label the endogenous F-actin. Scale 10 μm. **(D)** Quantification data showing that the percentage of F-actin positive cilia number (F-actin positive cilia number/total cilia number*100) was increased in the BBS group compared to the WT and *Ift88*^{OSNKO} group. One way ANOVA, *p < 0.05, ****p<0.0001, Values represent means ± SEM. **(F)** The relative F-actin positive cilia length (F-actin positive cilia length/ full cilia length*100) was increased in the BBS group compared to the WT and *Ift88*^{OSNKO} group. One way ANOVA, ****p<0.0001, Values represent means ± SEM

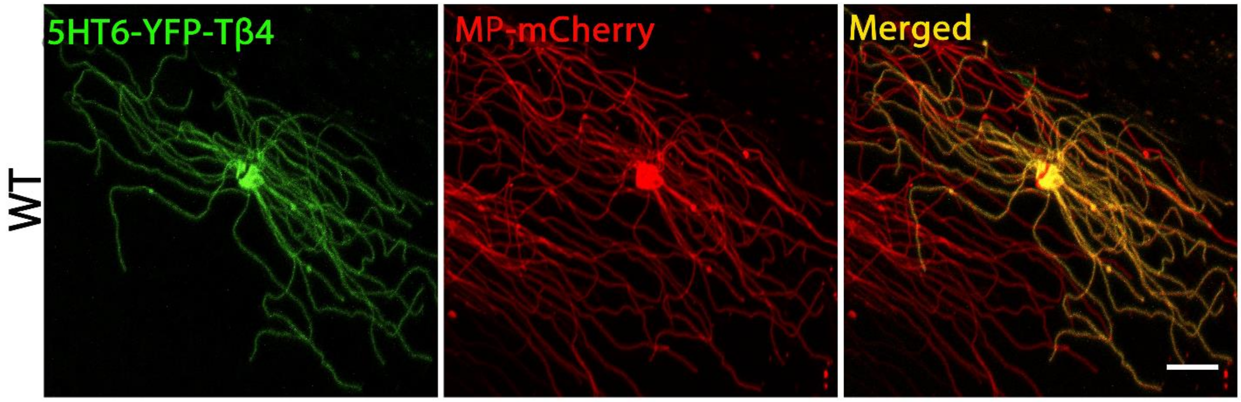

Supplementary Figure 5. Ectopically expressed 5HT6-YFP-Thymosinβ4 distributes to the full length of the olfactory cilia. Representative *en face* confocal microscopy images of ectopically co-expressing 5HT6-YFP-Thymosinβ4 and MP-mCherry in the WT OSNs. MP-mCherry was used to label the full length of the olfactory cilia. Scale 10 μm.

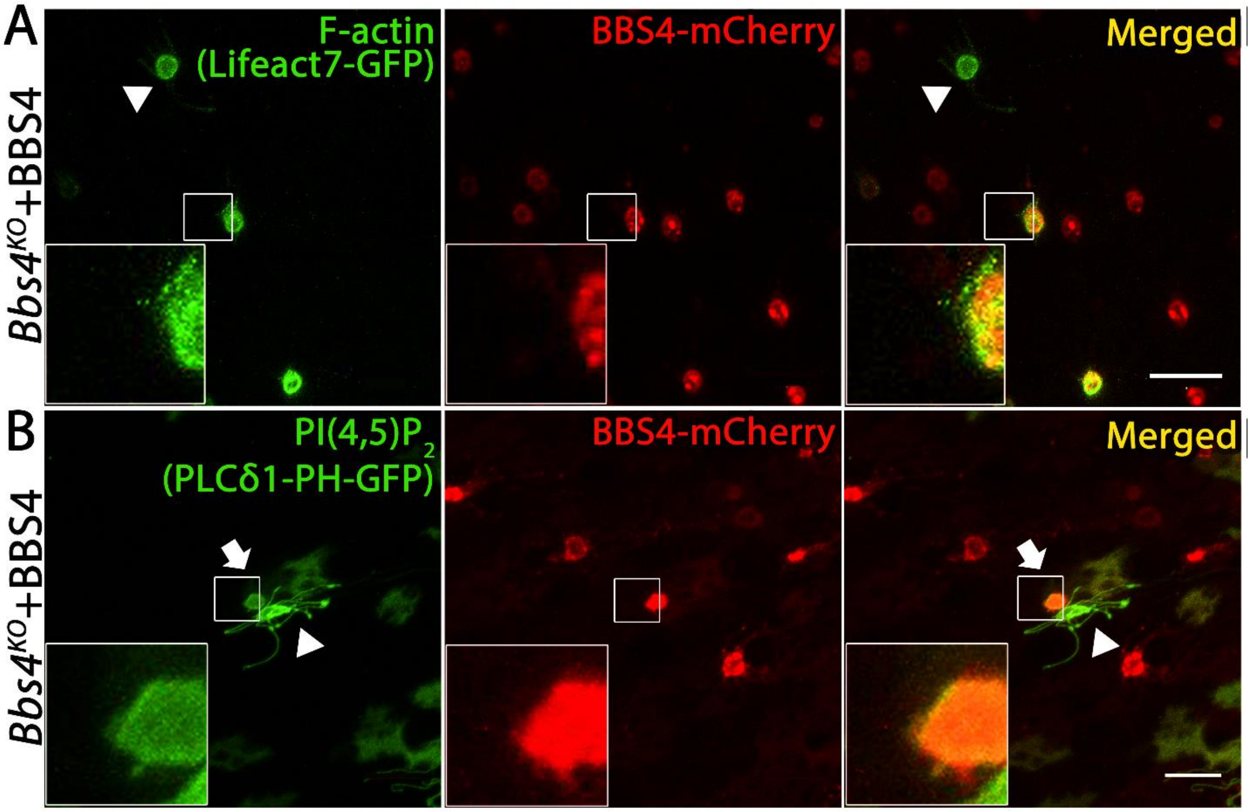

Supplemental Figure 6. Normal F-actin and PI(4,5)P_2 ciliary distribution is restored by *Bbs4* single gene replacement in *Bbs4^{KO}*. (A) Representative *en face* images of F-actin (Lifeact7-GFP) in BBS4 rescued *Bbs4^{KO}* olfactory cilia. F-actin redistributed into the olfactory cilia in non-BBS4 expressed OSN (arrowheads). *Bbs4* single gene replacement excludes F-actin from olfactory cilia in *Bbs4^{KO}* mice. Scale 10 µm. **(B)** *En face* images of PI(4,5)P_2 (PLCδ1-PH-GFP) in *BBS4* rescued *Bbs4^{KO}* OSN cilia. The expression of BBS4-mCherry blocked PI(4,5)P_2 ciliary distribution (arrow). Arrowhead indicating the non-BBS4-mCherry infected OSN still had PI(4,5)P_2 ciliary redistribution. Scale 10 µm.
